# The anti-tumor NC1 domain of collagen XIX inhibits the FAK/ PI3K/Akt/mTOR signaling pathway through αvβ3 integrin interaction

**DOI:** 10.18632/oncotarget.6399

**Published:** 2015-11-26

**Authors:** Jean-Baptiste Oudart, Manon Doué, Alexia Vautrin, Bertrand Brassart, Christèle Sellier, Aurelie Dupont-Deshorgue, Jean-Claude Monboisse, François-Xavier Maquart, Sylvie Brassart-Pasco, Laurent Ramont

**Affiliations:** ^1^ Université de Reims Champagne-Ardenne, CNRS UMR 7369 (Matrice Extracellulaire et Dynamique Cellulaire, MEDyC), Reims, France; ^2^ CHU de Reims, Laboratoire Central de Biochimie, Reims, France

**Keywords:** collagen XIX, NC1 domain, integrin, FAK/PI3K/Akt/mTOR, tumor invasion

## Abstract

Type XIX collagen is a minor collagen associated with basement membranes. It was isolated for the first time in a human cDNA library from rhabdomyosarcoma and belongs to the FACITs family (Fibril Associated Collagens with Interrupted Triple Helices). Previously, we demonstrated that the NC1 domain of collagen XIX (NC1(XIX)) exerts anti-tumor properties on melanoma cells by inhibiting their migration and invasion. In the present work, we identified for the first time the integrin αvβ3 as a receptor of NC1(XIX). Moreover, we demonstrated that NC1(XIX) inhibits the FAK/PI3K/Akt/mTOR pathway, by decreasing the phosphorylation and activity of the major proteins involved in this pathway. On the other hand, NC1(XIX) induced an increase of GSK3β activity by decreasing its degree of phosphorylation. Treatments targeting this central signaling pathway in the development of melanoma are promising and new molecules should be developed. NC1(XIX) seems to have the potential for the design of new anti-cancer drugs.

## INTRODUCTION

Collagen XIX was isolated from human rhabdomyosarcoma cells in 1992 by Hidei Yoshioka, under the name of collagen Y [1]. According to its amino acid sequence, it was classified in the FACIT family (Fibril Associated Collagens with Interrupted Triple helices). This initial classification was confirmed by several groups [2, 3]. In 1993, Myers et al. described a new collagen named RH COL [4] which turned out to be the same as collagen Y, so that, in 1994 the collagen XIX name replaced the two previous names [5]. Type XIX collagen has high homology with collagens IX, XII and XVI, in particular the presence of many well-conserved cysteine residues [6].

The type XIX collagen is a homotrimer of 400 kDa, composed of the combination of three 112 kDa α1(XIX) chains. Each chain is encoded by a 250 Kbp gene (encompassing 51 exons) located on chromosome 6 q12-q14 and consists of 1142 residues, with 5 collagenous domains (Col1 to Col5) interrupted by six non-collagenous domains (NC1 to NC6) [7, 8]. NC1(XIX) is a 19 amino acid peptide localized at the C-terminal end of the α1(XIX) chain. Unlike other FACIT collagens, α1(XIX) collagen chain has interchain and intrachain disulfide bonds which allow the formation of oligomers of different sizes [5, 8]. For our part, we have recently shown in the laboratory that the 19 amino acid NC1(XIX) forms intrachain disulfide bonds in solution [9].

Collagen XIX is ubiquitously expressed during embryogenesis where it is involved in the assembly of the extracellular matrix. In adults, its distribution is more restricted: vessels, skeletal muscle, spleen, prostate, kidney, liver, colon, placenta, breast, neurons and skin [10, 11]. It is involved in embryogenesis, in muscle differentiation [12] and in the development of esophagus [13]. Mice lacking collagen XIX are not viable and die early, due to abnormalities in muscle development. More recently, collagen XIX was reported to play a role in the hippocampal synapses formation [14].

In 2003, Myers et al. demonstrated that collagen XIX disappears early in the development of invasive breast cancer even before invasive stages [11]. This was the first description of the possible involvement of this collagen in the control of tumor invasion. By analogy with other NC1 domains of basement membrane collagens, we hypothesized that NC1(XIX) could have anti-tumor effects and demonstrated that it was able to inhibit *in vitro* migration and invasion of melanoma cells and inhibited melanoma growth *in vivo* [15, 16]. We also showed that plasmin, a key enzyme in tumor invasion, releases a peptide derived from the NC1 domain that has the same anti-tumor effects as the entire NC1 domain [9].

Integrins are transmembrane receptors often involved in tumor invasion. They are heterodimeric molecules composed of two subunits, α and β [17]. Anti-tumor matrikines were reported to bind integrin receptors but other receptor types are not excluded. For example, tumstatin (the NC1 domain of the α3 chain of collagen IV) binds the αvβ3 and α3β1 integrins [18, 19, 20], arresten (the NC1 domain of the α1 chain of collagen IV) binds the α1β1 integrin [21], canstatin (the NC1 domain of α2 chain of collagen IV) binds the αvβ3 and αvβ5 integrins [22], tetrastatin (the NC1 domain of the α4 chain of collagen IV) binds the αvβ3 integrin [23] and endostatin (the NC1 domain of the α1 chain of collagen XVIII) interacts with the α3β1, α5β1 and αVβ3 integrins [24, 25, 26].

Many signaling pathways are involved in the different stages of melanoma development, as reported by Uddensky et al. [27]. The FAK (Focal Adhesion Kinase) / PI3K (PhosphoInositide 3-Kinase) / Akt (proteine Kinase B) / mTOR (Mammalian Target Of Rapamycin) pathway is the most commonly involved signaling pathway. [28, 29]. This pathway is well characterized in melanoma carcinogenesis and involves a phosphorylation cascade well known in the literature [30] and often activated through integrin binding [27].

The aim of this work was to identify the receptor(s) of the NC1 (XIX) domain on melanoma cells and to identify the involved signaling pathway that could explain its anti-tumor effects in melanoma. We demonstrated the involvement of αvβ3 integrin and a decrease in the phosphorylation of the FAK/PI3K/Akt/mTOR signaling pathway.

## RESULTS

### NC1(XIX) inhibits the migration of SK-MEL-28 melanoma cells

**Figure 1:**
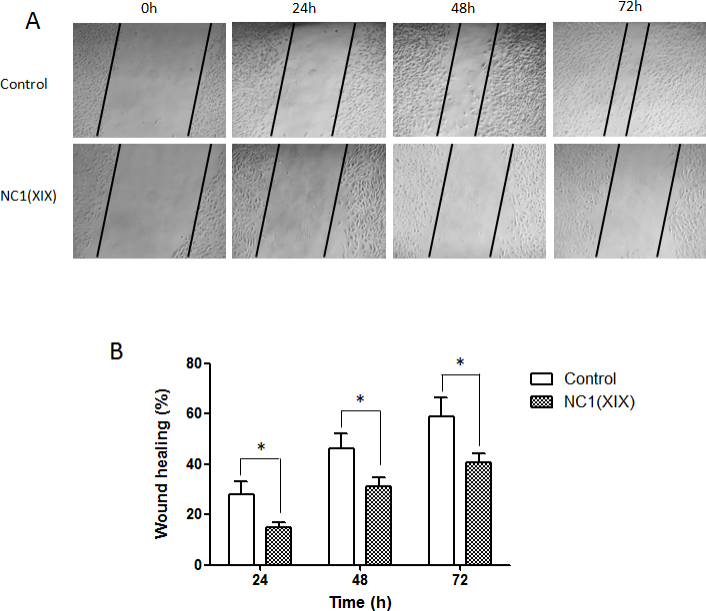
NC1(XIX) inhibits migration of SK-MEL-28 melanoma cells (**A**) SK-MEL-28 cells were seeded in 12-well plates. Cell layer was scratched with a pipet tip and then incubated with or without 20 μM of NC1(XIX) up to 72 h at 37°C. Wound closure was measured by microscopy at 0, 24, 48, 72 h respectively. (**B**) Wound closure was significantly delayed after NC1(XIX) treatment compared to control. Histogram represents the mean ± SEM of 4 replicates. *Significantly different from control (*p < 0.05).

Previous studies performed in our laboratory clearly demonstrated anti-tumor and anti-angiogenic activities of NC1(XIX). These results were confirmed on SK-MEL-28 melanoma cell line. In scratch wound assay, migration of SK-MEL-28 melanoma cells was significantly decreased after NC1(XIX) treatment (Figure 1A). NC1(XIX) inhibited melanoma cell migration by 47% (*p* < 0.05), 33% (*p* < 0.05) and 31% (*p* < 0.05) at 24, 48 and 72 h, respectively (Figure 1B).

### αvβ3 integrin specifically binds NC1(XIX)

**Figure 2:**
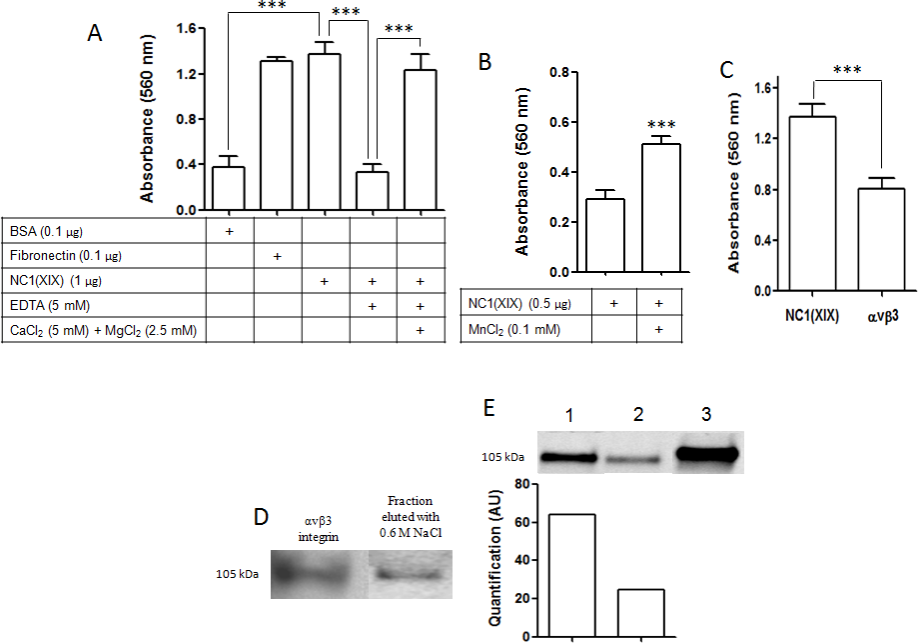
αvβ3 integrin specifically binds NC1(XIX) (**A**) Effect of divalent cations and EDTA on SK-MEL-28 cell adhesion. Cells were seeded in DMEM containing 5 mM CaCl_2_ + 2.5 mM MgCl_2_ and/or 5 mM EDTA, in wells coated with 0.1 μg BSA used as negative control, 0.1 μg fibronectin used as positive control or 1 μg of NC1(XIX) for 2 h at 37°C. (**B**) Effect of Mn^2+^ on SK-MEL-28 cell adhesion. Cells were seeded in HEPES buffer containing 0.1 mM Mn^2+^ in well coated with 1 μg of NC1(XIX) for 2 h at 37°C. (**C**) Effect of αvβ3 antibody on SK-MEL-28 cell adhesion. Cells were preincubated in DMEM containing 20 μg/mL of αvβ3 antibody for 30 min at 37°C and then incubated in wells coated with 1 μg of NC1(XIX) for 2 h at 37°C. After washing, cells were fixed with glutaraldehyde and stained with crystal violet. Absorbance was read at 560 nm. The values are the means ± SD of 4 replicates. *Significantly different from control (***p < 0.001). (**D**) SK-MEL-28 extracts were submitted by affinity chromatography on a NC1(XIX) peptide-bounded column. Bound proteins were eluted with increasing concentrations of NaCl (0.15, 0.6 and 1 M) and eluted samples were then analyzed by SDS-PAGE and western blot. The 0.6 M eluted sample revealed a band which matched the 105 kDa band of the denaturated recombinant αvβ3 integrin used as positive control. (**E**) In the liquid phase binding assay, recombinant αvβ3 integrin was incubated with NC1(XIX) biotinylated peptide with or without an excess of NC1(XIX) (lane 1 and 2 respectively). Integrin-peptide complexes were precipitated using sepharose streptavidin beads and revealed by western blot analysis. Recombinant αvβ3 integrin was used a positive control (lane 3).

Integrins have been demonstrated to serve as receptors of several matrikines, such as tumstatin or endostatin. Since integrins require divalent cations to bind ligands, we investigated the effects of Ca^2+^, Mg^2+^, Mn^2+^ and EDTA on cell adhesion to NC1(XIX) (Figure 2). SK-MEL-28 cell adhesion to NC1(XIX) was significantly increased compared to the negative control, BSA (*p* < 0.001), whereas adhesion was similar to the positive control, fibronectin. Adhesion of SK-MEL-28 cells was significantly decreased in the presence of 5 mM EDTA (*p* < 0.001). Cell adhesion was significantly restored when Ca^2+^ and Mg^2+^ were added into the media in order to counteract EDTA effect (*p* < 0.001) (Figure 2A). Likewise, SK-MEL-28 cell adhesion to NC1(XIX) was significantly increased in the presence of 0.1 mM Mn^2+^ (*p* < 0.001) (Figure 2B). Taken together, these results suggest the involvement of a cation-dependent receptor in SK-MEL-28 adhesion to NC1(XIX).

SK-MEL-28 melanoma cells were preincubated with monoclonal antibody directed against human αvβ3 integrin and then seeded in NC1(XIX) coated wells for 2 h at 37°C. The addition of the αvβ3 integrin monoclonal blocking antibody significantly decreased cell adhesion compared to control (*p* < 0.001) (Figure 2C), suggesting αvβ3 integrin as a potential receptor for NC1(XIX).

SK-MEL-28 extracts were analyzed by affinity chromatography on a NC1(XIX)-Sepharose column. Proteins bound to the affinity column were eluted with increasing concentrations of NaCl (0.15, 0.6 and 1.0 M). Eluted samples were submitted to SDS-PAGE and analyzed by western blot. The 0.6 M eluted samples revealed a band which matched the 105 kDa band of the denaturated recombinant αvβ3 integrin (Figure 2D). This result suggested an interaction between NC1(XIX) and αvβ3 integrin.

The interaction was confirmed in liquid phase binding assay. Recombinant αvβ3 integrin was incubated with biotinylated NC1(XIX) peptide with or without excess of unlabeled NC1(XIX) peptide. Integrin-peptide complexes were precipitated using sepharose-streptavidin beads and revealed by western blot. As shown in Figure 2E, biotinylated NC1(XIX) peptide specifically binds recombinant αvβ3 integrin (lane 1). The binding is reduced in the presence of unlabeled NC1(XIX) peptide (lane 2). Recombinant αvβ3 integrin was used as positive control (lane 3).

### NC1(XIX) colocalizes with αvβ3 integrin in SK-MEL-28 melanoma cells

**Figure 3:**
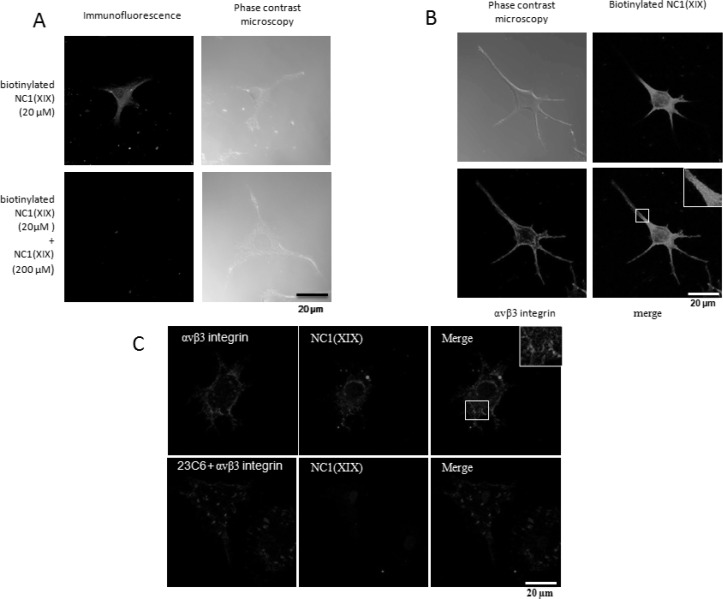
NC1(XIX) colocalizes with the αvβ3 integrin in SK-MEL-28 melanoma cells (**A**) Fluorescent microscopy visualization of biotinylated NC1(XIX) on the cell surface of SK-MEL-28 melanoma cells (top panel). Competition between NC1(XIX) and biotinylated NC1(XIX) (low panel). (**B**) Fluorescent microscopy visualization of biotinylated NC1(XIX) (green) and αvβ3 integrin (red). Yellow staining corresponds to areas where biotinylated NC1(XIX) and anti-αvβ3 integrin colocalize. Cells were cultured on glass slides, fixed with paraformaldehyde and labeled with biotinylated NC1(XIX) peptide and anti-αvβ3 integrin antibody. Scale bar: 20 μm. (**C**) SK-MEL-28 melanoma cells were cultured on glass slides, fixed with paraformaldehyde and incubated with biotinylated NC1(XIX) peptide and anti-αvβ3 integrin antibody (23C6).

SK-MEL-28 melanoma cells were cultured on glass slides, fixed with paraformaldehyde and incubated with biotinylated NC1(XIX) peptide, unlabeled NC1(XIX) peptide. Immunofluorescence studies showed biotinylated NC1(XIX) peptide (20 μM) (green) fixation on SK-MEL-28 cell surface. Competition assay with 10 fold-concentrated NC1(XIX) peptide (200 μM) completely abolished the biotinylated NC1(XIX) peptide labeling on SK-MEL-28 (Figure 3A). These data demonstrated the specificity of the NC1(XIX) peptide immunofluorescence fixation. As shown in Figure 3B, immunocytofluorescence microscopy analysis showed colocalization (yellow staining) between biotinylated NC1(XIX) peptide (green) and αvβ3 integrin (red) which suggested that αvβ3 integrin may be one of the NC1(XIX) SK-MEL-28 receptors.

Moreover, SK-MEL-28 melanoma cells were cultured on glass slides, fixed with paraformaldehyde and incubated with biotinylated NC1(XIX) peptide and anti-αvβ3 integrin antibody (23C6). Competition assay with anti-αvβ3 integrin antibody (23C6) abolished the biotinylated NC1(XIX) peptide labeling on SK-MEL-28 (Figure 3C).

### cRGDfV blocking peptide inhibits the NC1(XIX) binding on αvβ3 integrin

**Figure 4:**
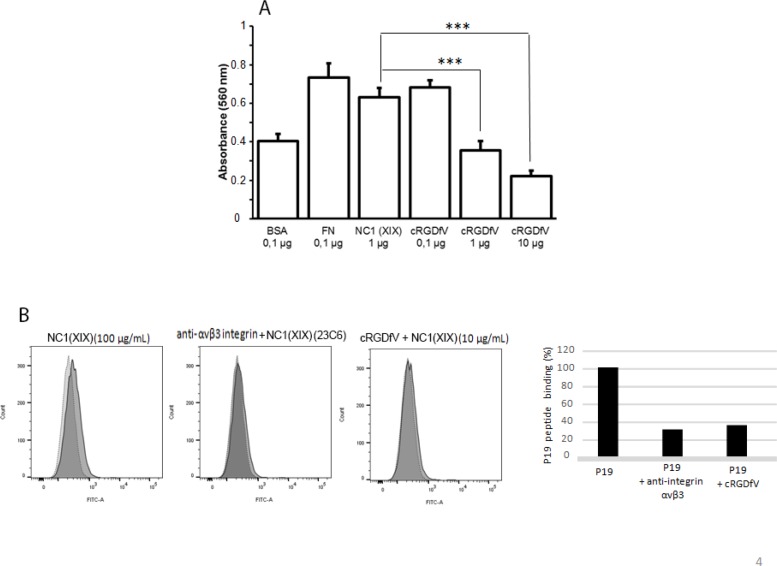
cRGDfV blocking peptide inhibits the NC1(XIX) binding on αvβ3 integrin (**A**) Effect of cRGDfV blocking peptide on SK-MEL-28 cell adhesion. Cells were preincubated in DMEM containing 0.1, 1 or 10 μg/mL of cRGDfV blocking peptide for 30 min at 37°C and then incubated in wells coated with 1 μg of NC1(XIX) for 2 h at 37°C. After washing, cells were fixed with glutaraldehyde and stained with crystal violet. Absorbance was read at 560 nm. The values are the means ± SD of 4 replicates. *Significantly different from control (***p < 0.001). (**B**) Flow cytometry studies showed NC1(XIX) peptide (100 μM) binding on SK-MEL-28 cell surface. (**C**) NC1(XIX) peptide (100 μM) binding was inhibited by the addition of αvβ3 integrin antibody (LM609) or cRGDfV blocking peptide.

SK-MEL-28 melanoma cells were preincubated with cRGDfV αvβ3 blocking peptide and then seeded in NC1(XIX)-coated wells for 2 h at 37°C. The addition of cRGDfV blocking peptide significantly decreased cell adhesion compared to control (*p* < 0.001) (Figure 4A).

We characterized the expression of integrins at the cell surface and demonstrated the expression of the integrin αvβ3 on the cells surface using flow cytometry (Supplementary Figure 1) and by immunocytofluorescence microscopy analysis (Supplementary Figure 2). Moreover, flow cytometry studies showed NC1(XIX) peptide (100 μM) fixation on SK-MEL-28 cell surface. This binding was inhibited by the addition of cRGDfV blocking peptide or anti-αvβ3 integrin antibody (23C6) (Figure 4B and 4C).

### NC1(XIX) decreases FAK^Y861^ phosphorylation, PI3K P85^Y458^ phosphorylation and PDK1^S241^ phosphorylation in SK-MEL-28 melanoma cells

**Figure 5:**
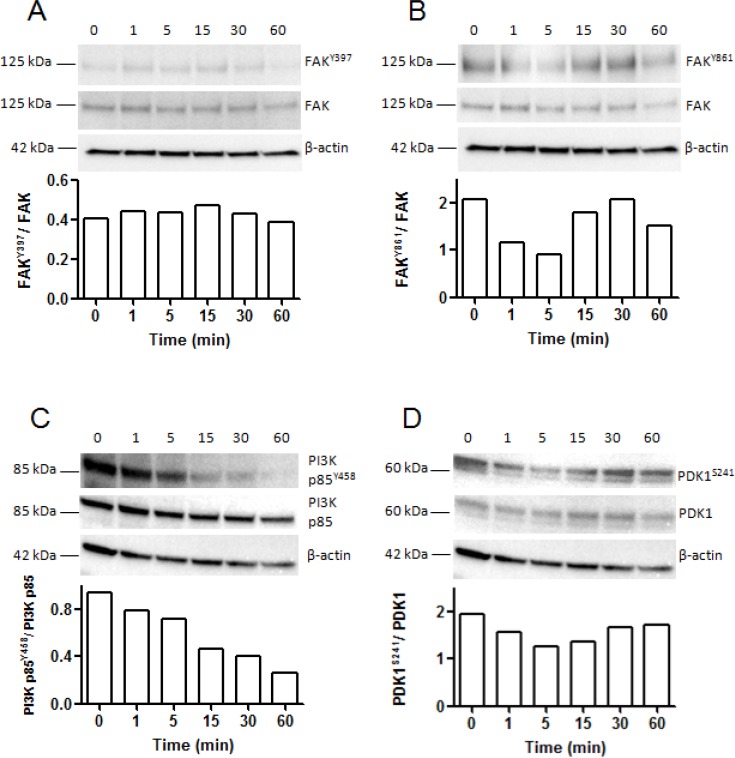
Kinetic analysis of FAK, PI3K p85 subunit of PI3 kinase and PDK1 phosphorylation in SK-MEL-28 melanoma cells after incubation with NC1(XIX) Western blot analysis of: (**A**) phosphorylated-FAK^Y397^, (**B**) phosphorylated-FAK^Y861^, (**C**) phosphorylated-PI3K p85^Y458^ and (**D**) phosphorylated-PDK1^S241^ compared to total FAK, total PI3K p85 and total PDK1 expression respectively after incubation of SK-MEL-28 melanoma cells with NC1(XIX) for 0, 1, 5, 15, 30 and 60 min. Bands were quantified by densitometric analysis. Phosphorylated protein was reported to corresponding total protein.

It is now well established that the FAK/PI3K/Akt/mTOR pathway plays a critical role in the development of melanoma. SK-MEL-28 melanoma cells were incubated with NC1(XIX) for 1, 5, 15, 30 and 60 min. Expression of total protein and correspondent phosphorylated protein were evaluated by western blot. FAK^Y397^ phosphorylation was not modified even after 60 min of incubation with NC1(XIX). Total FAK level and FAK^Y397^ / total FAK ratio remained also unchanged (Figure 5A). By contrast, as shown in Figure 5B, FAK^Y861^ phosphorylation decreased gradually after 1 min and 5 min of incubation with NC1(XIX), while total FAK level remained unchanged. The FAK^Y861^ / total FAK ratio was decreased by 50% at 5 min and was progressively restored after 30 min. In the same conditions, Tyr458 phosphorylation of the PI3K p85 subunit decreased gradually from 1 min to 60 min of incubation, while total PI3K p85 level remained unchanged. The PI3K p85^Y458^ / total PI3K p85 ratio was decreased by 20% at 5 min and 70% at 60 min (Figure 5C). In the same way, NC1(XIX) induced a decreased in PDK1^S241^ phosphorylation after 1 and 5 min of incubation, while total PDK1 level remained unchanged. The PDK1^S241^ / total PDK1 ratio was decreased by 30% at 5 min and remained decreased for at least 60 min (Figure 5D).

### NC1(XIX) decreases Akt^T308^ phosphorylation, Akt^S473^ phosphorylation, mTOR^S2448^ phosphorylation, mTOR^S2481^ phosphorylation and GSK3β^S9^ phosphorylation in SK-MEL-28 melanoma cells

**Figure 6:**
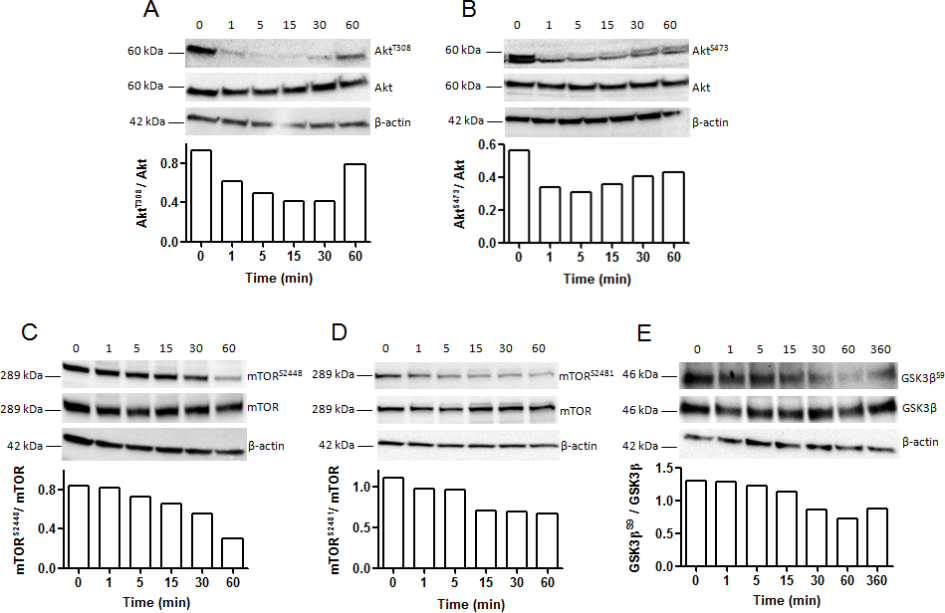
Kinetic analysis of Akt, mTOR and GSK3β phosphorylation in SK-MEL-28 melanoma cells after incubation with NC1(XIX) Western blot analysis showing expression of: (**A**) phosphorylated-Akt^T308^, (**B**) phosphorylated-Akt^S473^, (**C**) phosphorylated-mTOR^S2448^, (**D**) phosphorylated-mTOR^S2481^ and (**E**) phosphorylated-GSK3β^S9^ compared to total Akt, total mTOR and total GSK3β expression, respectively, after incubation of SK-MEL-28 melanoma cells with NC1(XIX) for 0, 1, 5, 15, 30, 60 and 360 min. Bands were quantified by densitometric analysis. Phosphorylated protein was reported to corresponding total protein.

Akt is located at the crossroads of numerous signaling pathways and plays a key role in many biological activities. NC1(XIX) induced a decrease in Akt^T308^ phosphorylation from 1 to 30 min of incubation, while total Akt level remained unchanged. The Akt^T308^ / total Akt ratio decreased of 20% at 1 min and 50% at 15 and 30 min (Figure 6A). In the same way, NC1(XIX) also triggered a decrease in Akt^S473^ phosphorylation. The Akt^S473^ / total Akt ratio decreased of 30% at 60 min (Figure 6B). As shown in Figure 6C, mTOR^S2448^ phosphorylation decreased gradually from 15 min to 60 min of incubation with NC1(XIX), while total mTOR level remained unchanged. The mTOR^S2448^ / total mTOR ratio was decreased by 25% at 30 min and 60% after 60 min of incubation. In the same way, NC1(XIX) induced a decreased in mTOR^S2481^ phosphorylation after 15 min. The mTOR^S2481^ / total mTOR ratio also decreased of 30% from 15 to 60 min of incubation (Figure 6D).

In the same conditions, Ser 9 phosphorylation of GSK3β decreased gradually from 30 min to 60 min of incubation, while total GSK3β level remained unchanged. The GSK3β^S9^ / total GSK3β ratio was gradually decreased by 30% at 30 min, 40% at 60 min and remained decreased by 30% at 360 min (Figure 6E).

## DISCUSSION

Our previous studies demonstrated that the NC1 domain of collagen XIX had anti-tumor effects by inhibiting migration and invasion of melanoma cells without affecting their proliferation [15, 16]. We also showed that plasmin, a key enzyme in tumor invasion, generates a peptide derived from NC1(XIX) which reproduces the same inhibiting effects as the entire NC1(XIX) domain on the tumor cells *in vitro* and *in vivo* [9]. The aim of this study was to highlight the receptor(s) involved in the anti-tumor effect of NC1(XIX) and to elucidate the elicited signaling pathway.

Our present results demonstrated that the NC1(XIX) inhibits the migration of SK-MEL-28 cells. These results confirm previous studies from our laboratory demonstrating anti-tumor activity of NC1(XIX) [15].

We showed that the adhesion of NC1(XIX) on SK-MEL-28 melanoma cells was calcium/magnesium/manganese dependent, evoking the involvement of integrins. Integrins play key roles in malignant process as well in the early stages of tumor invasion as in the tumor angiogenesis or in the development of metastases [31]. We identified for the first time αvβ3 integrin as a receptor for NC1(XIX) by affinity chromatography and liquid phase assay. This integrin is particularly involved in tumor genesis and was broadly described as one of the common receptor of the basement membrane collagens [32]. In confocal microscopy, we showed a co-localization of the integrin and NC1(XIX), showing proximity and interaction between these two molecules on melanoma cells. Furthermore, the zone of binding between the NC1(XIX) peptide and integrin αvβ3 is close to the integrin region of interaction with RGD peptide. This was demonstrated by the competition found between the NC1(XIX) peptide and RGD peptide. Moreover, other receptors are probably involved since the inhibition observed was 50% only when blocking αvβ3 integrin.

FAK is a multifunctional protein [33], especially involved in tumor invasion [29], angiogenesis [34] and energy metabolism of the tumor cell [35]. This is one of the molecules activated after ligand fixation [36]. Its activity is the result of a complex phosphorylation cascade leading to the activation of multiple proteins in the tumor process. Activation begins by auto-phosphorylation on tyrosine 397 following the integrin liganding [29]. Later on, the auto-phosphorylation allows the recruitment of Src protein which in turn phosphorylates FAK on many tyrosine residues (Tyr407, Tyr576, Tyr577, Tyr861, and Tyr925) [29, 37].

FAK^Y861^ phosphorylation has been well studied in tumors and it is clear that this phosphorylation is essential for tumor cell migration [38]. We demonstrated that phosphorylation of FAK^Y861^ was inhibited by NC1(XIX) as soon as 1 min of incubation. Afterwards, this inhibition is the starter of a series of FAK partner phosphorylation inhibitions, which may explain the anti-tumor and anti-angiogenic effects of NC1(XIX).

Two main signaling pathways, the RAS /RAF/MEK/ERK pathway and the FAK/PI3K/Akt/mTOR pathway are activated in melanoma and may explain the progression of this cancer [39]. After activation of FAK *via* integrins, the PI3K/Akt/mTOR pathway is involved in the proliferation, migration, and angiogenesis of many cancers, including melanoma [39, 40].

Phosphorylation by the PI3K activating subunit is important, especially for the migration and growth of tumor cells [41, 42]. Our results show that NC1(XIX) inhibits the phosphorylation of the regulatory subunit p85 of PI3K on tyrosine 458. Activated FAK was reported to phosphorylate the downstream PI3K that, in turn, activates Akt [43]. Akt is a central enzyme in tumor invasion, which participates in the regulation of all stages of tumor development [44]. Inhibition of PI3K and Akt promoted anoikis and decreased melanoma tumor growth by inhibiting the Rho subfamily [45]. PDK1 (phosphoinositide-dependent kinase 1) and the complex mTORC2 activates Akt through threonine 308 and serine 473 phosphophylations, respectively [43, 46].

As mentioned above, these two Akt-activating protein kinases are also activated by phosphorylation, essential for their activity, on serine 241 for PDK1 [47]and on serine 2481 for mTORC2 [48].

We demonstrated that NC1(XIX) inhibited the phosphorylation of many partners (Akt^S473^, Akt^T308^, PDK1^S241^, mTORC2^S2481^) of the signaling pathway. In addition, NC1(XIX) also induced an inhibition of phosphorylation of the second mTORC1 complex on Serine 2448. This phosphorylation is involved in tumor invasion, particularly in melanoma progression [49] and its phosphorylation is essential for its activity [48, 50].

Moreover, we demonstrated that NC1(XIX) induced an inhibition of GSK3β (Glycogen synthase kinase 3-β) by decreasing its phosphorylation degree. GSK3β plays an important role in the activity of mTOR complex [51]. It is a serine/threonine kinase initially described in the regulation of glycogen synthesis [52]. It also plays a significant role on directional cell migration [53] and is described as a therapeutic target in melanoma [54]. Unlike most kinases, GSK3β is activated under its dephosphorylated form. More recently, Koo et al. have shown that activation of GSK3β was essential for potentiating the action of anti-tumoral mTOR inhibitor [55]. The increase of GSK3β activity induced by NC1(XIX), through a GSK3β dephosphorylation process, could explain the effects of NC1(XIX) on mTOR inhibition and could also explain its anti-tumor effects on SK-MEL-28 and other melanoma cell lines previously described [15, 16].

GSK3β implication in the mechanism of action of NC1 (XIX) suggests the involvement of the Wnt / β-catenin signaling pathway. This pathway is frequently deregulated in carcinogenesis and is most often of poor prognosis, in particular in colon cancer [56]. However, its role in melanoma is still controversial and ambiguous. The Wnt / β-catenin pathway is sometimes pro-tumoral and sometimes anti-tumoral in other circumstances. According to the literature, it inhibits the proliferation and migration of tumor cells but increases their metastatic potential [57–58]. Given the complexity of this pathway and its role in melanoma, studying the effects of NC1 (XIX) on this pathway will be part of our prospects.

**Figure 7:**
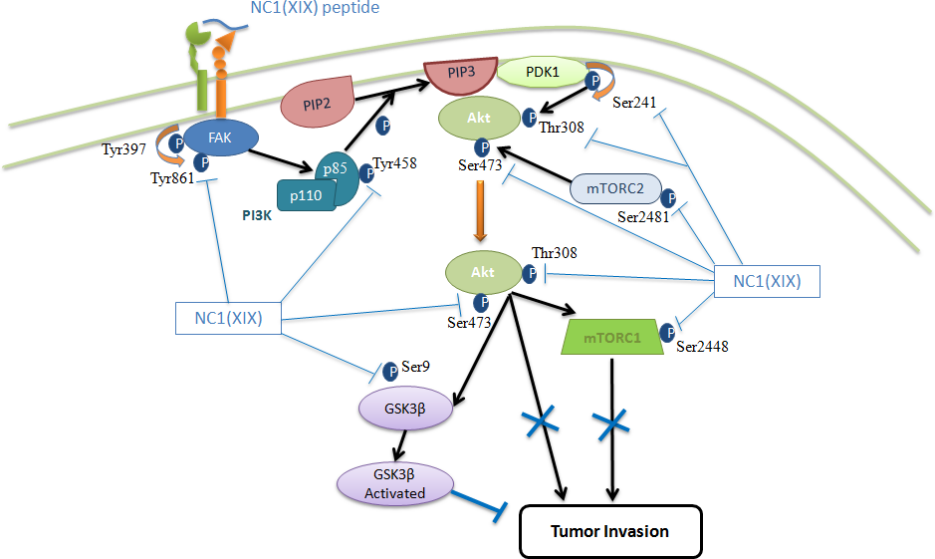
Schematic representation of the FAK / PI3K / Akt / mTOR pathway and modifications of phosphorylated proteins after incubation with NC1(XIX)

In summary, we have identified for the first time the αvβ3 integrin as a receptor of the NC1 domain of the collagen XIX. We have demonstrated that NC1(XIX) controls the majority of the partners of the FAK/PI3K/Akt/mTOR pathway by inducing the inhibition of their phosphorylations, essential for their biological activity (Figure 7). Inhibition of several participants in a signaling pathway is more powerful than that of a single molecule [55]. Treatments targeting this central signaling pathway in the development of melanoma are promising [30] and new molecules should be developed in this in this aim. NC1(XIX) seems to have the potentiality for the design of new anti-cancer drugs.

## MATERIALS AND METHODS

### Peptides synthesis

NC1(XIX) peptide was purchased from Proteogenix^®^ (Schiltigheim, France). It was obtained by solid-phase synthesis using a FMOC (N-(9-fluorenyl)methoxy-carbonyl) derivative procedure. It was then purified by reverse phase high performance liquid chromatography using a C18 column, eluted by a gradient of acetonitrile in trifluoroacetic acid, and finally lyophilized [59]. The NC1(XIX) peptide sequence was NPEDCLYPVSHAHQRTGGN. Biotinylated NC1(XIX) peptide was also purchased from Proteogenix^®^ and had the following sequence NPEDCLYPVSHAHQRTGGN-K-Biotin.

### Cell culture

SK-MEL-28 melanoma cell line was purchased from the American Type Culture Collection (ATCC, Manassas, VA). Cell were grown in Dulbecco's Modified Eagle's medium (DMEM) supplemented with 10% Fetal Bovine Serum (FBS) in Nunclon^®^ 25 cm^2^, 75 cm^2^ or 150 cm^2^ flasks (Dutscher Brumath, France) at 37°C in a humid atmosphere (5% CO_2_, 95% air). All culture media and reagents were purchased from Life Technologies (Invitrogen, Strasbourg, France).

### Adhesion assay

For cell adhesion assays, 96-well microtiter plates were purchased from Dutscher (Brumath, France). Plates were coated with the different substrates described below, diluted in carbonate buffer (0.2 M sodium carbonate, 0.2 M sodium bicarbonate, pH 9.6) overnight at 4°C. Bovine serum albumin (BSA) (Euromedex, Souffelweyersheim, France) at 0.1 μg/well was used as negative control whereas fibronectin (Millipore, Molsheim, France) at 0.1 μg/well was used as positive control. NC1(XIX) was coated in the same carbonate buffer at 1 μg/well. Cells were detached with a Versene buffer (126 mM NaCl, 5 mM KCl, 1 mM EDTA, 50 mM HEPES) and centrifuged at 800 g for 5 min at room temperature. Cells were then incubated with 2 mM CaCl_2_ and 0.5 mM MgCl_2_ in DMEM. After counting, cells were seeded at a density of 60,000 cells in 100 μL/well and incubated for 2 h at 37°C in 5% CO_2_. Unattached cells were removed by washing with PBS. Attached cells were fixed with 1.1% glutaraldehyde in PBS for 20 min. After washing, cells were stained with 0.1% crystal violet for 20 min. After extensive washing with distilled water and air-drying, crystal violet was eluted in 100 μL/well of 10% acetic acid. Absorbance was then measured at 560 nm. To investigate the effect of divalent cations or EDTA on adhesion, cells were incubated for 2 hours at 37°C in 5% CO_2_ with or without 5 mM EDTA and/or 5 mM CaCl_2_ and 2.5 mM MgCl_2_ or 0.1 mM Mn^2+^. For MnCl_2_ adhesion assay, cells were centrifuged at 800 g for 5 min at room temperature and then incubated in HEPES buffer (150 mM NaCl, 50 mM HEPES). For adhesion blocking assay, cells were preincubated with 20 μg/mL of anti-αvβ_3_ integrin antibody (sc7312 from Santa Cruz Biotechnology, USA) diluted in 2 mM CaCl_2_ and 0.5 mM MgCl_2_ supplemented DMEM for 30 min on a carrousel at 37°C before adhesion assay. In complementary experiments, cells were incubated with 0.1 to 10 μg of cRGDfV (Calbiochem^™^) (blocking αvβ_3_ integrin peptide).

### Scratch wound assay

SK-MEL-28 cells were seeded in 12-well plates and cultivated to confluence in DMEM supplemented with 10% FBS at 37°C in 5% CO_2_. At confluence, cells were incubated with fresh DMEM without FBS overnight at 37°C. A homogenous wound was then created in each well with a sterile 200 μL pipet tip. After washing with DMEM, cells were incubated in DMEM with or without 20 μM NC1(XIX) for 24, 48 or 72 h at 37°C. Wounds were microphotographed at 0, 24, 48 or 72 h of incubation and wound closure was measured.

### Immunocytofluorescence assay

30,000 SK-MEL-28 melanoma cells were seeded on coverslip placed into 24-well culture plate and then incubated for 24 h in DMEM supplemented with 10% FBS at 37°C in 5% CO_2_. Cells were fixed with 4% paraformaldehyde in PBS (w/v) and incubated for 10 min at room temperature. After washing with PBS, cells were then incubated with biotinylated NC1(XIX), NC1(XIX) and/or anti-αvβ3 integrin antibody (MAB1976 Millipore, Molsheim, France) diluted 1/200 in 1% BSA-supplemented PBS for 1 h at room temperature. After washing with PBS, cells were incubated respectively with a 1/1000 diluted Alexa fluor 488- or 568-conjugated secondary antibody (A11057 and A11004 respectively, Invitrogen, Carlsbad, USA) in PBS supplemented with 1% BSA for 30 min in a dark chamber at room temperature. Coverslips were then mounted with aqueous mounting medium (Thermo Scientific, Villebon sur Yvette, France) and examined using a confocal laser scanning microscope (Zeiss LSH710, Carl Zeiss MicroImaging, GmbH, Germany).

### Affinity chromatography

SK-MEL-28 protein extracts were chromatographed at 4°C on a HiTrap NHS-activated Sepharose High Performance column (GE Healthcare, Orsay, France) previously functionalized with NC1(XIX) peptide, according to the manufacturer instructions. Unbound material was removed with 30 mL of washing buffer (10 mM Tris, 1 mM CaCl_2_, 1 mM MgCl_2_, pH 7.6, 1/100 PIC (ProteoBlock Protease Inhibitor Cocktail, Fermentas, Illkirch, France) (w/v) and 0.1% octylglucoside). Proteins bound to the affinity column were then eluted with elution buffer (10 mM Tris, pH 7.6, 1/100 PIC (w/v) and 0.1% octylglucoside) supplemented with increasing concentrations of NaCl (0.15, 0.6 and 1 M). Eluted samples were then solubilized in SDS sample buffer with 10 mM DTT, denatured at 95°C for 5 min and submitted to western blotting using an β3 integrin antibody (Santa Cruz Biotechnology, Heidelberg, Germany.

### Flow cytometry

Anti-integrin primary antibody and secondary anti-mouse or anti-rabbit antibody were used according to manufacturer's instruction (Supplementary Table 1). Cells were analysed by LSRFortessa flow cytometer (BD Biosciences) using a laser emitting at 488 nm and detecting Alexa Fluor 488 emission with a 530/30 bandpass filter. Ten thousand cells, gated on forward scatter vs. side scatter, were collected for each sample. Quantification of integrin expression was performed by median fluorescence intensity (MFI) ratio of sample / control. MFI ratio of 2 were used as the threshold for positivity. Results were analyzed using software Flow Jo (TreeStar, Inc; SA).

### Integrin binding assay

One μg of recombinant αvβ3 integrin (3050-AV-0505 R&D Systems) was combined with 1 μg of biotinylated NC1(XIX) peptide in 500 μL of Dulbecco's PBS without divalent cations (Ca^2+^ or Mg^2+^). This mixture was incubated with or without 7 μg of unbiotinylated NC1(XIX) peptide with gentle shaking at 4°C for 30 min. 10 μL of streptavidin sepharose beads (Cell Signaling Technology) were added to the samples for a second incubation period of 30 min at 4°C. Samples were washed twice with 1 mL PBS and centrifuged at 500 g for 5 min at room temperature. 20 μL of loading buffer (500 mM tris, 10% SDS (w/v), 20% saccharose (w/v), pH 6.8, 75 mg bromophenol blue, 5% 2-mercaptoethanol (v/v)) were added to each pellet. Samples were denatured at 95°C for 5 min, centrifuged and supernatants were submitted to SDS-PAGE.

### Western blotting

SK-MEL-28 melanoma cells were cultured as previously described in Nunclon 25 cm^2^ flasks. At 50%-confluence, cells were washed with PBS and incubated with DMEM without FBS overnight at 37°C. Cells were then treated with 50 μM NC1(XIX) diluted in DMEM without FBS for 0, 1, 5, 15, 30 or 60 min. All flasks were frozen at −80°C until protein extraction. Cells were scrapped with 200 μL of protein lysis buffer (RIPA buffer, Sigma, St Quentin Fallavier, France), supplemented with 1% PIC, 2 mM sodium orthovanadate and 50 mM NaF (Sigma, St Quentin Fallavier, France) and cell lysate were incubated for 30 min at 4°C. Cellular debris was pelleted by centrifugation of lysates at 10000 g for 10 min at 4°C. Protein concentration of the supernatant was quantified using Biorad Protein Assay (BioRad, Marnes-La-Coquette, France) according to the manufacturer's instructions.

For western blot analysis, samples were reduced by 10 mM dithiothreitol and denaturated 5 min at 95°C. Samples were submitted to SDS-PAGE (Criterion gel, BioRad, Marnes-La-Coquette, France) (20 μg of total protein per lane) and then transferred onto membrane using Transblot Turbo (BioRad, Marnes-La-Coquette, France). Membranes were blocked with 5% BSA in TBS-T (0.1% Tween 20, 50 mM Tris HCl buffer, 150 mM NaCl, pH 7.5) for 2 h at room temperature. All antibodies were incubated in TBS-T supplemented with 1% BSA overnight at 4°C. Antibody references and dilutions are summarized in Table 1. After washing with TBS-T, membranes were incubated with 1/10000 diluted corresponding peroxidase-conjugated second antibody diluted in TBS-T, 1% BSA for 1 h at room temperature. After washing, immune complexes were revealed using ECL prime chemiluminescence detection kit (GE Healthcare, Orsay, France) according to the manufacturer's instructions.

**Table 1:** Antibody list and dilution used

Antibody	Manufacturer	Reference	Dilution
Phospho-FAK^Tyr 458^	Novex by life Technology^™^	44626G	1/1000
Phospho-FAK^Tyr397^ (D20B1)	Cell Signaling Technology^®^	8556	1/1000
FAK	Cell Signaling Technology^®^	3285	1/1000
phospho-P13Kp85^Tyr458^	Cell Signaling Technology^®^	4228	1/1000
P13Kp85	Millipore^™^	06–195	1/1000
phospho-PDK1^Ser 241^(C49H2)	Cell Signaling Technology^®^	3438	1/1000
PDKI (D37A7)	Cell Signaling Technology^®^	5662	1/1000
phospho-AKT^Ser473^ (D9E)	Cell Signaling Technology^®^	4060	1/1000
phospho-AKT^Thr 308^ (C31E5E)	Cell Signaling Technology^®^	2965	1/1000
AKT	Cell Signaling Technology^®^	9272	1/1000
phospho-mTOR^Ser 2448^	Cell Signaling Technology^®^	2971	1/1000
phospho-mTOR ^Ser 2481^	Cell Signaling Technology^®^	2974	1/1000
mTOR (L27D4)	Cell Signaling Technology^®^	4517	1/1000
phospho-GSK3β^Ser 9^	Cell Signaling Technology^®^	9323	1/2000
GSK3β	Cell Signaling Technology^®^	9325	1/2000

### Statistical analysis

Results were expressed as means +/− standard deviation or SEM. Statistical significance between groups was assessed by unpaired Student's *t* test.

## SUPPLEMENTARY MATERIAL FIGURES AND TABLE


