# Supplementary material for: The anti-tumor NC1 domain of collagen XIX inhibits the FAK/ PI3K/Akt/mTOR signaling pathway through αvβ3 integrin interaction
Source: Oncotarget. 2015 Nov 26;7(2):1516–28. doi: 10.18632/oncotarget.6399 (PMC4811477; doi:10.18632/oncotarget.6399)
Supplement: Supplementary file 1 [file oncotarget-07-1516-s001.pdf]

The anti-tumor NC1 domain of collagen XIX inhibits the FAK/PI3K/Akt/mTOR signaling pathway through αvβ3 integrin interaction

Supplementary Materials

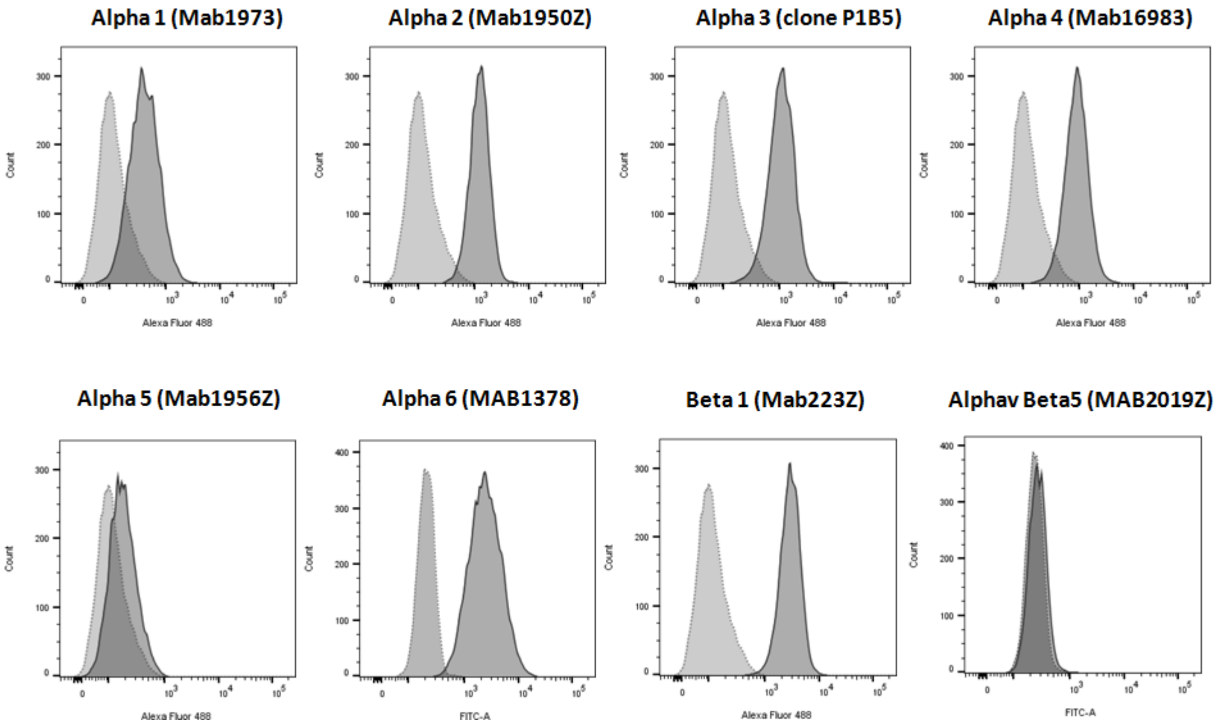

Supplementary Figure S1: Flow cytometry analysis of SKMEL28 cell surface integrin expression. SK-MEL28 cells express the α1, α2, α3, α4, α5, α6, αv, β1 and β3 integrin subunits but not β5 integrin subunit.

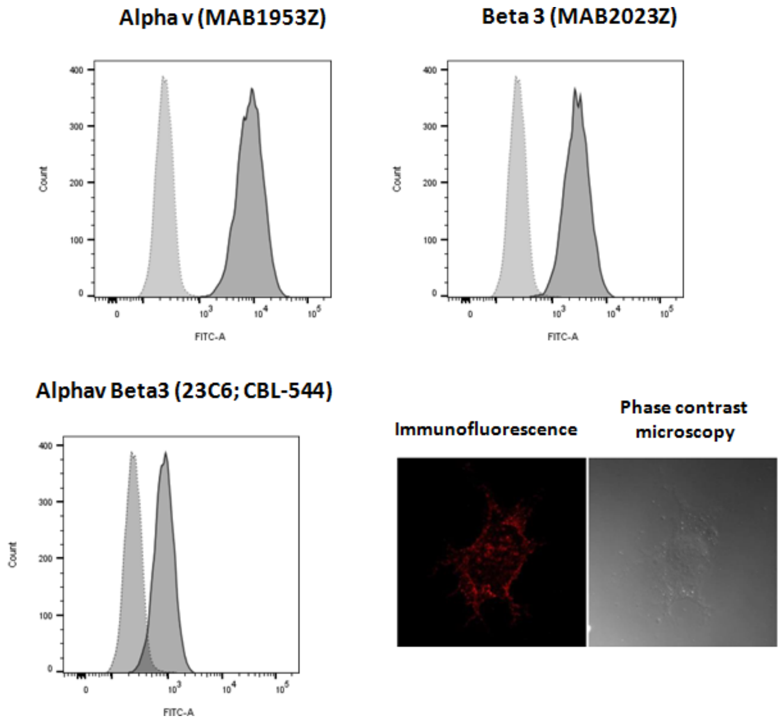

Supplementary Figure S2: Flow cytometry and immunocytofluorescence assay (confocal laser scanning microscope) analysis of SKMEL28 showing the expression of alpha v beta 3 integrin on the cell surface.

Supplementary Table S1: Anti-integrin primary antibody and secondary anti-mouse or anti-rabbit antibody used in flow cytometry experiments

Antibody	References	Manufacturers
Anti-Integrin α1	Mab1973	Millipore™
Anti-Integrin α2	Mab1950Z	Millipore™
Anti-Integrin α3	clone P1B5	Millipore™
Anti-Integrin α4	Mab16983	Millipore™
Anti-Integrin α5	Mab1956Z	Millipore™
Anti-Integrin α6	MAB1378	Millipore™
Anti-Integrin αv	MAB1953Z	Millipore™
Anti-Integrin β1	Mab223Z	Millipore™
Anti-Integrin β3	MAB2023Z	Millipore™
Anti-Integrin αvβ3	23C6; CBL-544	Millipore™
Anti-Integrinαvβ5	MAB2019Z	Millipore™
Streptavidin, Alexa Fluor® 488 conjugate	S-11223	Molecular Probes™
Goat anti-Mouse IgG<br>Alexa Fluor® 488 conjugate	A-11001	Molecular Probes™
Goat anti-Rat IgG<br>Alexa Fluor® 488 conjugate	A-11006	Molecular Probes™
